# High-flow nasal cannula versus noninvasive ventilation in acute heart failure–related respiratory failure: a systematic review and meta-analysis

**DOI:** 10.3389/fmed.2026.1941162

**Published:** 2026-09-09

**Authors:** Fatima Binte Athar, Abdulrahim Shujaa Almutairi, Mohamed Mohamed Basha, Ammar Khalid Alhajjaj, Tamir Hassan, Ammar Halawani, Mohammed Abdulrahman Alrashidi, Ahmed Hamdy G. Ali, Ali Bakr, Abdulrahman Yamani

**Affiliations:** 1 College of Medicine, Alfaisal University, Riyadh, Saudi Arabia; 2 College of Medicine, Qassim University, Buraidah, Saudi Arabia; 3 Department of Psychiatry, King Salman Medical City, Madinah, Saudi Arabia; 4 Dar Alsalam Medical Center, Ad Duwaimah, Madinah, Saudi Arabia; 5 College of Medicine, King Abdulaziz University Hospital, Jeddah, Saudi Arabia; 6 College of Medicine, Shaqra University, Shaqra, Saudi Arabia; 7 College of Medicine, National Research Mordovia State University, Institute of Medicine, Saransk, Russia; 8 College of Medicine, Al-Azhar University, Cairo, Egypt; 9 Department of Emergency, Al Iman General Hospital, Riyadh, Saudi Arabia

**Keywords:** acute heart failure, cardiogenic pulmonary edema, high-flow nasal cannula, noninvasive ventilation, respiratory failure

## Abstract

**Background:**

Noninvasive ventilation (NIV) is the recommended first-line respiratory support for acute heart failure (AHF)–related respiratory failure. However, its tolerance is often limited. High-flow nasal cannula (HFNC) has emerged as a potential alternative, but comparative evidence remains inconsistent. This study aimed to evaluate the efficacy and safety of HFNC versus NIV in patients with AHF-related respiratory failure.

**Methods:**

We conducted a systematic review and meta-analysis following PRISMA guidelines. We searched four major electronic databases from inception to April 2026. We included randomized controlled trials (RCTs) and observational studies enrolling adult patients with respiratory failure secondary to AHF or acute cardiogenic pulmonary edema, comparing HFNC and NIV. The primary outcome was composite treatment failure. Secondary outcomes included endotracheal intubation, short-term mortality, arterial blood gas (ABG) parameters, respiratory rate, dyspnea scores, and hospital length of stay. A random-effects model was used. The certainty of evidence was assessed using the Grading of Recommendations Assessment, Development and Evaluation (GRADE) approach.

**Results:**

Ten studies (*n* = 1,282 patients) were included, comprising five RCTs and five observational studies. HFNC showed no statistically significant difference compared with NIV in the composite outcome of treatment failure (RR = 1.22, 95% CI: 0.81–1.84), endotracheal intubation (RR = 0.86, 95% CI: 0.58–1.28), or short-term mortality (RR = 0.99, 95% CI: 0.69–1.42). Design-stratified estimates for treatment failure also crossed the line of no effect in both RCTs (RR = 1.00, 95% CI: 0.61–1.63) and observational studies (RR = 1.44, 95% CI: 0.77–2.69). No statistically significant differences were observed in ABG parameters, respiratory rate, dyspnea scores, or hospital length of stay. The GRADE assessment showed predominantly moderate certainty evidence for RCT comparisons, with high-certainty evidence for changes in PaCO_2_ and pH.

**Conclusion:**

The available evidence did not find a statistically significant difference between HFNC and NIV in terms of clinical efficacy in patients with AHF-related respiratory failure. HFNC may be considered in selected patients who do not tolerate NIV, provided that close monitoring and prompt access to NIV escalation are available. Although the certainty of evidence was generally moderate for RCTs comparisons, further adequately powered multicenter RCTs are needed to better define patient selection and clinically important differences.

**Systematic review registration:**

www.crd.york.ac.uk/PROSPERO/view/CRD420261330512, identifier CRD420261330512.

## 1 Introduction

Acute heart failure (AHF) represents a severe cardiovascular emergency and remains a leading etiology for hospital admissions among older adults, accounting for approximately 20% of all emergency department visits for acute respiratory failure (1, 2). The condition carries a substantial prognostic burden, demonstrating an in-hospital mortality rate approaching 10% alongside a high incidence of early hospital readmission (3).

Current international cardiology and respiratory guidelines strongly recommend noninvasive ventilation (NIV), encompassing both continuous positive airway pressure (CPAP) or bilevel positive airway pressure (BiPAP), as the standard first-line respiratory support strategy (4). NIV effectively improves arterial oxygenation, decreases respiratory muscle workload, and optimizes cardiac hemodynamics by reducing left ventricular afterload and right ventricular preload through intrathoracic positive pressure (5, 6). Despite these well-documented physiological benefits, up to 40% of patients experience poor tolerance to tight-fitting NIV masks due to claustrophobia, facial skin breakdown, or patient-ventilator asynchrony, frequently resulting in treatment failure and the subsequent need for invasive mechanical ventilation (7).

High-flow nasal cannula (HFNC) oxygen therapy presents a better-tolerated, patient-centered alternative (8). HFNC delivers heated and humidified oxygen, generates a flow-dependent positive end-expiratory pressure, and facilitates continuous anatomical dead-space washout to improve carbon dioxide clearance (9, 10). While the physiological rationale supports HFNC application, existing primary trials comparing HFNC against NIV in AHF or acute cardiogenic pulmonary edema (ACPE) continue to report discordant clinical outcomes regarding overall treatment failure, intubation rates, and rapid respiratory rate reduction (10).

Furthermore, previous meta-analyses had methodological limitations, including heterogeneous non-cardiogenic respiratory-failure populations, earlier search dates, or absence of recently published trials (11, 12). We therefore conducted an updated meta-analysis restricted to adults with AHF-related respiratory failure, evaluating the comparative efficacy and safety of HFNC versus NIV.

## 2 Methods

We conducted and reported this systematic review and meta-analysis in accordance with the Preferred Reporting Items for Systematic Reviews and Meta-Analyses (PRISMA) guidelines and Cochrane handbook of systematic reviews (13, 14). We registered the study protocol prospectively on the International Prospective Register of Systematic Reviews (PROSPERO) under the registration number: [CRD420261368165].

### 2.1 Eligibility criteria

We defined our study eligibility using the PICOS framework. We included studies enrolling adult patients (≥18 years old) with acute respiratory failure attributable to AHF or ACPE, or with established heart failure receiving prophylactic HFNC or NIV immediately after planned extubation. Eligible studies evaluated the use of HFNC oxygen therapy, including all high-flow oxygen delivery systems, compared with NIV, specifically CPAP or BiPAP. We considered both randomized controlled trials (RCTs) and observational studies for inclusion.

We excluded studies involving mixed populations when heart-failure-specific outcome data could not be extracted separately. Additionally, we excluded case reports, editorials, narrative reviews, and animal studies.

### 2.2 Information sources and search strategy

Two independent researchers (F.B.A and A.S.A) systematically searched four electronic databases: PubMed, Scopus, Web of Science, and the Cochrane Library, from inception until April 2026. We formulated the search strategy using a combination of Medical Subject Headings (MeSH) and free-text keywords. The search terms primarily included variations of “Heart Failure,” “Pulmonary Edema,” “High-Flow Nasal Cannula,” and “Noninvasive Ventilation.” We applied no language or publication date restrictions. Supplementary Table 1 outlines the detailed search strategy applied to each database.

### 2.3 Study selection

Following the database search, we exported all retrieved citations into the Rayyan web application to identify and remove duplicate records (15). Two reviewers independently (M.M.B and A.K.A) screened the titles and abstracts of the remaining articles to assess their initial eligibility. Subsequently, the reviewers retrieved the full texts of potentially eligible studies and evaluated them against our predefined inclusion criteria. The reviewers resolved any disagreements during the selection process through direct discussion, and when necessary, consulted a third senior author to reach a final consensus.

### 2.4 Data extraction

Two independent reviewers (T.H and A.H) extracted data from the final included studies into a standardized spreadsheet. We extracted the following variables: study characteristics (first author, publication year, study design, country, setting, and follow-up duration), patient baseline demographics (sample size, age, sex, comorbidities, initial vital signs, and blood gas parameters), and treatment details (initial settings for both HFNC and NIV). We resolved any data extraction discrepancies through cross-checking and mutual agreement.

### 2.5 Risk of bias assessment

We evaluated the methodological quality of the included studies based on their design. Two independent reviewers (M.A.A and A.B) assessed the risk of bias for RCTs using the Cochrane Risk of Bias 2 (RoB-2) tool, which evaluates five domains: bias arising from the randomization process, deviations from intended interventions, missing outcome data, measurement of outcomes, and selection of the reported results (16).

For observational studies, two independent reviewers (F.B.A and M.M.B) assessed study quality using the Newcastle–Ottawa Scale (NOS), which assesses study quality based on three domains: selection of participants, comparability of study groups, and ascertainment of outcomes/exposures (17).

Any disagreements were resolved through discussion or consultation with a third reviewer (A.S.Y).

### 2.6 Certainty of evidence

The certainty of evidence for each outcome was independently assessed by two reviewers (AB and AHGA) using the Grading of Recommendations Assessment, Development and Evaluation (GRADE) approach (18). Randomized controlled trials started as high-certainty evidence, whereas observational studies started as low-certainty evidence. The certainty of evidence was rated down according to the risk of bias, inconsistency, indirectness, imprecision, and publication bias, and was rated up when predefined GRADE criteria were fulfilled. Because randomized and observational studies were analyzed separately in predefined subgroup analyses, certainty ratings were also reported separately for each body of evidence. Disagreements were resolved through discussion until consensus was reached.

### 2.7 Outcome measures

The primary outcome of this meta-analysis was the composite of treatment failure. Definitions varied across studies and could include intubation, mortality, crossover or escalation because of intolerance or lack of efficacy, or an explicitly reported treatment-failure outcome. The study-specific definitions of treatment failure across the included studies are summarized in Supplementary Table 2.

Secondary clinical outcomes included the tracheal intubation rate and short-term mortality. We defined short-term mortality as the closest reported timepoint in each study, including in-hospital, ICU, 28-day, or 30-day mortality. Furthermore, we assessed physiological and symptomatic outcomes, including changes from baseline in Arterial Blood Gas (ABG) parameters (PaO_2_, PaCO_2_, and pH), changes from baseline in RR, dyspnea scores, and hospital length of stay (measured in days).

### 2.8 Statistical analysis

We performed all statistical analyses using Stata software, version 19. For dichotomous outcomes, we calculated Risk Ratios (RR) with their corresponding 95% Confidence Intervals (CI). For continuous outcomes, we calculated Mean Differences (MD) with 95% CIs. We applied a random-effects model across all analyses to account for anticipated clinical and methodological variations among the included studies.

We assessed statistical heterogeneity using the I^2^ statistic and the Cochran Q test, defining an I^2^ value greater than 50% as indicative of moderate to high heterogeneity.

To explore potential sources of heterogeneity, we conducted subgroup analyses stratified by study design (RCTs versus observational studies).

We also performed a leave-one-out sensitivity analysis to assess the influence of individual studies on the overall pooled estimate. Additionally, we performed a sensitivity analysis excluding the post-extubation cohort and the highly unbalanced observational cohort to evaluate their influence on the primary outcome.

Finally, we constructed funnel plots to visually inspect for publication bias and used Galbraith plots to identify specific outlier studies contributing to statistical heterogeneity.

## 3 Results

### 3.1 Literature search

**FIGURE 1:**
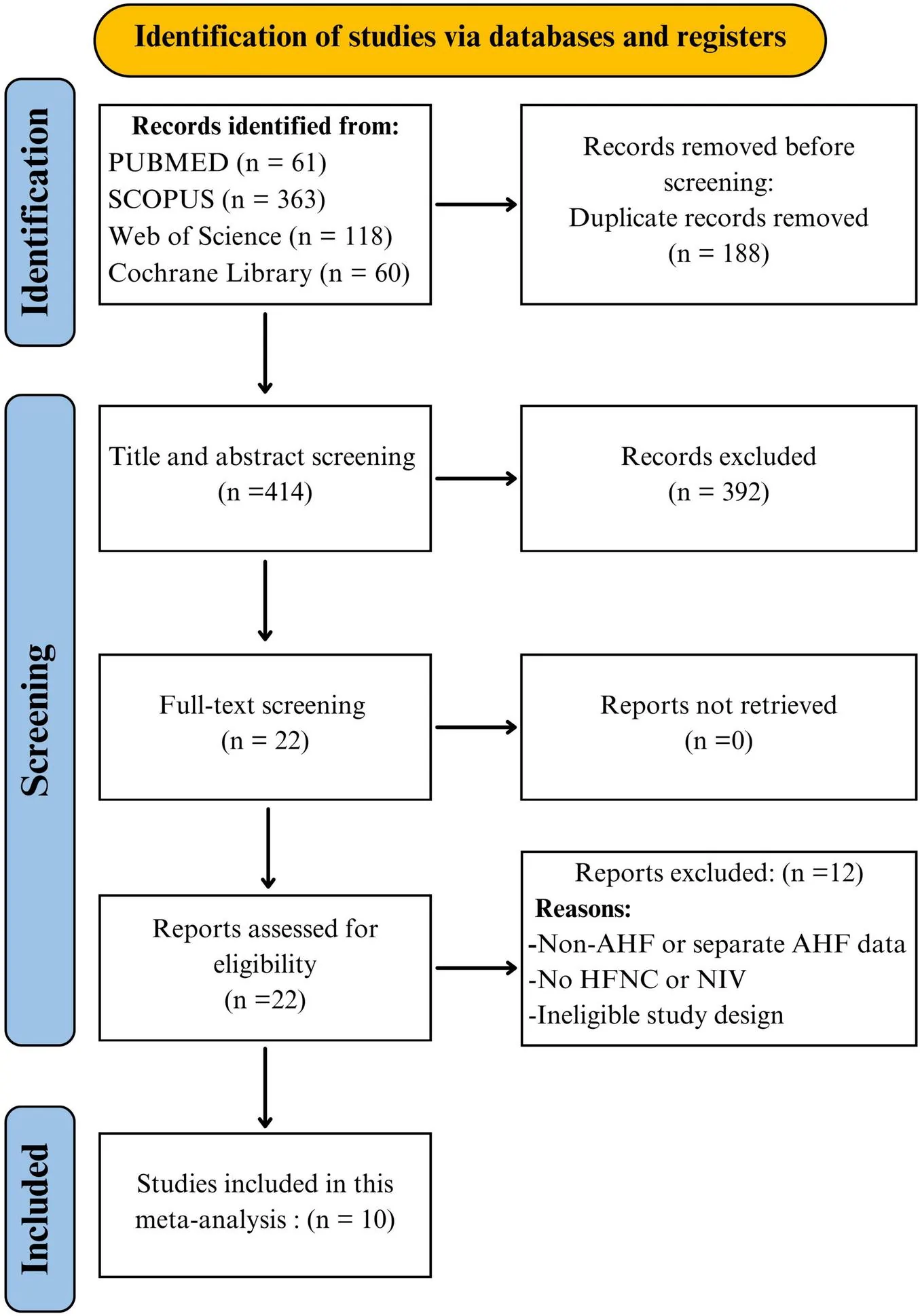
PRISMA flow diagram of study selection process. Flow diagram illustrating the process of study identification, screening, eligibility, and inclusion. PRISMA, preferred reporting items for systematic reviews and meta-analyses.

We retrieved a total of 602 records from the initial database search. After using Rayyan to remove 188 duplicate records, we screened the titles and abstracts of the remaining 414 articles. We excluded 392 records at this stage. Subsequently, we sought and successfully retrieved the full texts for 22 reports to assess their final eligibility. We excluded 12 reports for the following reasons: inclusion of non-AHF populations or failure to report separate AHF data, lack of HFNC or NIV interventions, and ineligible study designs. Finally, we included 10 studies in this systematic review and meta-analysis. The PRISMA flow diagram is presented in Figure 1.

### 3.2 Characteristics of included studies

This study included 10 studies published between 2019 and 2025, comprising a total of 1,282 adult patients (10, 19–27). The included studies consisted of five RCTs (10, 20, 21, 25, 27) and five observational studies (19, 22–24, 26). The investigators conducted these studies across various countries, including France, Turkey, the USA, Brazil, Malaysia, Taiwan, Spain, Japan, and China. Patient enrollment primarily occurred in emergency departments (EDs) and intensive care units (ICU).

The included populations covered acute AHF/ACPE-related respiratory failure across emergency and critical-care settings, separately reported cardiac subgroups from broader acute respiratory failure cohorts, and one post-extubation cohort of patients with established heart failure. For the intervention, initial HFNC settings varied, generally utilizing flows between 30 and 60 Liters Per Minute (LPM) with titrated fractions of inspired oxygen (FiO_2_). The comparator group received NIV, encompassing CPAP, including helmet CPAP in one trial, or BiPAP.

Baseline characteristics revealed an older patient demographic, with median or mean ages typically exceeding 60 years and reaching up to 91 years in some cohorts. Common comorbidities among the participants included hypertension, diabetes mellitus, ischemic heart disease, and COPD. Baseline vital signs and blood gas parameters reflected severe acute respiratory distress at presentation across both treatment arms.

Tables 1, 2 detail the individual study characteristics and baseline population data.

**TABLE 1:** Summary of included studies.

Study ID	Sample size	Study design	Country	Setting	Population	Inclusion criteria	High-flow initial settings	NIV initial settings	Follow-up duration (Days)	Conclusion
Altunbas 2025 (27)	178	Single-center RCT	Turkey	ED (Single center)	ACPE	Clinical ACPE; RR > 24; SpO_2_ < 92% on room air; WOB signs.	60 LPM; 31 °C; FiO_2_ titrated for SpO_2_ ≥ 92%.	CPAP; PEEP 5–10; FiO_2_ titrated for SpO_2_ ≥ 92%.	0.08	HFNC is as effective as NIV in reducing RR and symptoms, it also provides better tolerability and comfort.
Haywood 2019 (25)	42	RCT	USA	ED (Multicenter)	RF secondary to ADHF	Adults in ED; clinical need for NIPPV; discharge diagnosis of ADHF.	35 LPM; 35–37 °C; FiO_2_ 1.0; titrated to target.	BiPAP; IPAP 10; EPAP 5; FiO_2_ 1.0; titrated to target.	3	HFNC is non-inferior to NIV regarding intubation rates and treatment success for ADHF patients.
Maia 2024 (RENOVATE) (20)	272	Multicenter RCT	Brazil	ED/ICU/Ward (Multicenter)	ACPE (Subgroup of ARF)	Adults; ARF; clinical diagnosis of ACPE.	45–60 LPM; FiO_2_ titrated for SpO_2_ 92–98%.	IPAP 12–14; EPAP 8; FiO_2_ titrated for SpO_2_ 92–98%.	90	HFNC is non-inferior to NIV regarding intubation or death at 7 days.
Marjanovic 2024 (10)	60	Multicenter RCT	France	ED (Multicenter)	ACPE	RR ≥ 25 or signs of increased WOB; clinical suspicion of ACPE.	Min 50 LPM; 37 °C; FiO_2_ 0.50; titrated for SpO_2_ ≥ 94%.	Bi-level; PS 8; PEEP 5; FiO_2_ 0.50; titrated for SpO_2_ ≥ 94%.	28	No significant difference in RR change between groups. HFNC may improve comfort.
Osman 2021 (21)	206	Single-center RCT	Malaysia	ED (Single center)	ACPE	Older than 18 years; RR > 30; SpO_2_ < 90% on ≥ 15L O_2_; clinical ACPE diagnosis.	50 LPM (start); 37°C; FiO_2_ titrated for SpO_2_ > 94%.	Helmet CPAP; PEEP 5; FiO_2_ 0.60; SpO_2_ > 94%.	28	Helmet CPAP achieved greater short-term improvement in RR and hemodynamics than HFNC.
Chang 2020 (26)	104	Prospective observational	Taiwan	ICU (Single center)	HF (LVEF < 50%) post-extubation	HF with LVEF < 50%; ETT > 24 h; passed weaning protocol.	49.4 LPM; FiO_2_ 41.9%.	IPAP 13.4; PEEP 7.3; FiO_2_ 35.1%.	72	HFNC is non-inferior to NPPV for preventing reintubation in HF patients.
Hinojosa 2021 (24)	47	Prospective observational	Spain	CCU (Single center)	Non-hypercapnic APE	Clinical APE; PaCO_2_ ≤ 45 mmHg; clinician discretion for therapy.	44.3 LPM; 37 °C.	CPAP (7 cmH_2_O) or NIPPV (14/6 cmH_2_O).	30	HFNC was not associated with increased mortality but showed a trend toward more therapy failures.
Koga 2020 (23)	190	Multicenter retrospective	Japan	ED/ICU (Multicenter)	CPE (Subgroup of ARF)	Adults; ARF with P/F ratio < 300; CPE as primary diagnosis.	40 LPM; FiO_2_ 0.80.	EPAP 6 cmH_2_O; FiO_2_ 0.60.	30	In the ACPE subgroup, HFNC was associated with a higher risk of treatment failure vs. NIV.
Marjanovic 2020 (22)	27	Prospective observational	France	ED (Single center)	Hypercapnic RF due to CPE	Signs of ARF; RR ≥ 20; PaCO_2_ > 45 mmHg; clinical CPE suspicion.	60 LPM; 37 °C; FiO_2_ titrated for SpO_2_ ≥ 92%.	Bi-level; PEEP 5–10; PS 6–10; FiO_2_ titrated for SpO_2_ ≥ 92%.	0.042–0.063	HFNC improves PaCO_2_ and RR in hypercapnic ACPE comparable to NIV.
Tong 2022 (19)	156	Retrospective cohort	China	ICU (Single center)	AHF with hypoxemia	Older than 18 years; history of HF or heart disease; PaO_2_ < 80 mmHg; SpO_2_ < 90%; LVEF < 50% or NYHA > II; BNP > 100.	30–40 LPM; 37 °C; FiO_2_ 40–100%; adjusted for SpO_2_ > 90%.	Bi-level; IPAP 8; EPAP 4; RR 15–20	9	Both HFNC and NIV significantly improved physiological parameters and cardiac function within 24 h of initiation. However, the HFNC group achieved greater amelioration compared to NIV.

ACPE, acute cardiogenic pulmonary edema; RR, respiratory rate; SpO_2_, peripheral oxygen saturation; WOB, work of breathing; LPM, liters per minute; FiO_2_, fraction of inspired oxygen; CPAP, continuous positive airway pressure; PEEP, positive end-expiratory pressure; HFNC, high-flow nasal cannula; NIV, non-invasive ventilation; RCT, randomized controlled trial; ED, emergency department; RF, respiratory failure; ADHF, acute decompensated heart failure; NIPPV, non-invasive positive pressure ventilation; BiPAP, bilevel positive airway pressure; IPAP, inspiratory positive airway pressure; EPAP, expiratory positive airway pressure; ARF, acute respiratory failure; ICU, intensive care unit; HF, heart failure; LVEF, left ventricular ejection fraction; ETT, endotracheal tube; NPPV, non-invasive positive pressure ventilation; APE, acute pulmonary edema; CCU, coronary care unit; PaCO_2_, partial carbon dioxide; CPE, cardiogenic pulmonary edema; P/F ratio, ratio of arterial oxygen partial pressure to fractional inspired oxygen; PS, pressure support; NR, not reported; AHF, acute heart failure; PaO_2_, partial oxygen; NYHA, New York Heart Association; BNP, B-natriuretic peptide.

**TABLE 2:** Baseline characteristics of populations in the included studies.

Study ID	Arm	Sam-ple	Age (years), mean (SD)	Male, *n* (%)	RR (breaths/min), mean (SD)	HR (beats/ min), mean (SD)	SBP (mm Hg), mean (SD)	DBP (mm Hg), mean (SD)	SpO_2_ (%), mean (SD)	PaO_2_ (mm Hg), mean (SD)	PaCO_2_ (mm Hg), mean (SD)	pH, mean (SD)	PaO_2_/ FiO_2_ ratio, mean (SD)	LVEF (%), mean (SD)	APACHE II score, mean (SD)	Dys pnea score, mean (SD)	Hyper tensio n (%)	DM, n (%)	IHD, n (%)	COPD, n (%)
Marjanovic 2020 (22)	HFNC	12	91 (77–94) *	7 (58.3)	34 (27–41) *	97 (80–128) *	156 (124–172) *	80 (63–85) *	93 (90–98) *	99 (61–155) *	50 (49–61) *	7.30 (7.20–7.36) *	NR	NR	NR	NR	5 (41.7)	2 (16.7)	NR	0 (0.0)
	NIV	15	82 (79–92) *	3 (20.0)	29 (26–36) *	79 (68–98) *	146 (111–184) *	71 (61–92) *	96 (94–98) *	102 (72–125) *	60 (48–71) *	7.29 (7.21–7.35) *	NR	NR	NR	NR	10 (66.7)	3 (20.0)	NR	0 (0.0)
Haywood 2019 (25)	HFNC	22	64.5 (59–77) *	5 (22.7)	33 (28–38) *	93 (84–101) *	NR	NR	95.5 (93–100) *	82 (60–162) *	42.5 (32–55) *	7.38 (7.27–7.45) *	118 (60–166) *	NR	31.5 (30–37) *	6 (3–9) *	NR	NR	NR	NR
	NIV	20	60 (53–71.5) *	10 (50.0)	34 (28–36) *	106 (92–110) *	NR	NR	98.5 (94–100) *	116.5 (81–283.5) *	42 (39–49) *	7.33 (7.32–7.38) *	153.5 (81.8–283.5) *	NR	29 (28–34) *	7 (5–9) *	NR	NR	NR	NR
Maia 2024 (RENOVATE) (20)	HFNC	136	71 (13)	70 (51.5)	28 (26–29) *	90 (73–105) *	NR	NR	88 (85–90) *	80 (62–106) *	37 (31–43) *	7.39 (7.34–7.44) *	247 (167–340) *	NR	NR	NR	104 (76.5)	64 (47.1)	NR	26 (19.1)
	NIV	136	70 (14)	68 (50.0)	28 (26–30) *	88 (73–107) *	NR	NR	88 (85–90) *	79 (60–107) *	40 (34–47) *	7.38 (7.31–7.42) *	225 (156–346) *	NR	NR	NR	117 (86.0)	69 (50.7)	NR	33 (24.3)
Marjanovic 2024 (10)	HFNC	30	85 (79–88) *	15 (50.0)	30.5 (28–33) *	82 (70–93) *	146 (130–167) *	74 (68–89) *	95 (92–97) *	84 (48–117) *	51 (22–68) *	7.32 (7.24–7.42) *	195 (146–269) *	51 (40–60) *	NR	NR	NR	8 (26.7)	NR	2 (6.7)
	NIV	30	88 (83–94) *	12 (40.0)	29.5 (27–35) *	81 (72–94) *	153 (137–182) *	81 (68–94) *	96 (93–97) *	83 (71–108) *	49 (39–60) *	7.33 (7.25–7.42) *	248 (146–286) *	50 (38–57) *	NR	NR	NR	14 (46.7)	NR	2 (6.7)
Tong 2022 (19)	HFNC	80	69.8 (7.4)	33 (41.3)	28 (7)	110 (29)	NR	NR	NR	49.4 (10.7)	53.1 (20.2)	7.1 (0.1)	144 (111–160) *	41.8 (5.0)	18.9 (4.4)	3.60 (0.5)	49 (61.3)	30 (37.5)	NR	21 (26.3)
	NIV	76	69.1 (6.9)	29 (38.2)	28 (6)	109 (26)	NR	NR	NR	49.6 (11.2)	52.8 (19.9)	7.1 (0.1)	148 (120–159) *	40.9 (4.8)	18.9 (4.6)	3.6 (0.5)	44 (57.9)	31 (40.8)	NR	21 (27.6)
Altunbas 2025 (27)	HFNC	90	70.5 (64–78)*	41 (45.6)	34 (30–38)*	NR	NR	NR	89 (85–91)*	NR	54 (50–61)*	7.3 (7.27–7.31)*	NR	NR	NR	7 (6–9)*	86 (95.6)	53 (58.9)	NR	35 (38.9)
	NIV	88	68 (60–75)*	37 (42.0)	33.5 (30–37)*	NR	NR	NR	90 (85–92)*	NR	54 (47–60)*	7.29 (7.27–7.31)*	NR	NR	NR	7 (6–9)*	79 (89.8)	45 (51.1)	NR	36 (40.9)
Hinojosa 2021 (24)	HFNC	28	67 (14.74)	24 (85.7)	27.74 (3.23)	94.2 (25.5)	117.5 (25.31)	68.2 (12.87)	89.8 (5.1)	NR	NR	NR	NR	39.9 (11.9)	15.05 (7.11)	NR	19 (67.9)	9 (32.1)	9 (32.1)	2 (7.1)
	NIV	19	70.42 (11.56)	15 (78.9)	28.4 (5.7)	103.1 (27.7)	125.3 (37.04)	75.1 (20.6)	89.6 (5.6)	NR	NR	NR	NR	33.8 (9.7)	15.34 (5.31)	NR	17 (89.5)	13 (68.4)	7 (36.8)	4 (21.1)
Osman 2021 (21)	HFNC	105	60.5 (57.3–63.6)^§^	63 (67.0)	33 (32–35)^§^	111 (108–113)^§^	NR	NR	NR	91.37 (83.88–98.85)^§^	36.62 (34.49–38.75)^§^	7.38 (7.36–7.40)^§^	152 (140–164)^§^	NR	NR	5 (2–10)*	65 (69.1)	55 (58.5)	21 (22.3)	NR
	NIV	101	60.9 (58.3–63.4)^§^	60 (63.8)	35 (34–36)^§^	112 (109–115)^§^	NR	NR	NR	87.26 (83–92)^§^	36.45 (34.21–38.70)^§^	7.35 (7.33–7.37)^§^	145 (138–153)^§^	NR	NR	6 (2–10)*	75 (79.8)	68 (72.3)	22 (23.4)	NR
Chang 2020 (26)	HFNC	58	74.5 (63.8–82.3)*	37 (63.8)	NR	NR	NR	NR	NR	NR	NR	NR	NR	33.55 (26.6–41.2)*	21 (16.8–27)*	NR	39 (67.2)	36 (62.1)	27 (46.6)^‡^	9 (15.5)^†^
	NIV	46	72.5 (61.8–79)*	28 (60.9)	NR	NR	NR	NR	NR	NR	NR	NR	NR	35.4 (27.9–41.1)*	24 (18–30)*	NR	37 (80.4)	22 (47.8)	19 (41.3)^‡^	11 (23.9)^†^
Koga 2020 (23)^‡‡^	HFNC	24	NR	NR	NR	NR	NR	NR	NR	NR	NR	NR	NR	NR	NR	NR	NR	NR	NR	NR
	NIV	166	NR	NR	NR	NR	NR	NR	NR	NR	NR	NR	NR	NR	NR	NR	NR	NR	NR	NR

RR, respiratory rate; HR, heart rate; SBP, systolic blood pressure; DBP, diastolic blood pressure; SpO_2_, peripheral oxygen saturation; PaO_2_, partial oxygen; PaCO_2_, partial carbon dioxide; pH, potential of hydrogen; PaO_2_/FiO_2_, ratio of arterial oxygen partial pressure to fraction of inspired oxygen; LVEF, left ventricular ejection fraction; APACHE II, acute physiology and chronic health evaluation II; DM, diabetes mellitus; IHD, ischemic heart disease; COPD, chronic obstructive pulmonary disease; HFNC, high-flow nasal cannula; NIV, non-invasive ventilation, and NR, not reported.

*Reported as median (interquartile range), not converted.

^§^ Reported as mean (95% confidence interval), not converted.

† Reported as Obstructive lung disease.

‡ Reported as coronary artery disease.

^‡‡^Koga et al. 2020 study evaluated a mixed acute respiratory failure population. While it specifies the total number of patients in the Cardiogenic Pulmonary Edema subgroup (*n* = 24 HFNC, *n* = 166 NIV), the study does not report baseline characteristics specific to this subgroup.

### 3.3 Risk of bias assessment

We assessed the five RCTs using the Cochrane RoB-2 tool. Overall, two studies were judged as low risk of bias, two studies had some concerns, and one study was rated as high risk of bias. The main limitation across trials was the lack of blinding, which likely influenced intervention adherence and outcome assessment (Supplementary Figure 1).

Regarding observational studies assessed using the NOS tool, the overall quality was predominantly good, with four studies rated as good quality and one study rated as poor quality (Supplementary Table 3).

### 3.4 Certainty of evidence

The certainty of evidence, assessed using the GRADE approach, varied across outcomes (Supplementary Table 4).

In the RCT-only analyses, the certainty of evidence was high for changes in PaCO_2_ and pH, moderate for composite treatment failure, tracheal intubation, short-term mortality, changes in PaO_2_, respiratory rate, and dyspnea score.

In the combined analyses of RCTs and observational studies, the certainty of evidence was moderate for tracheal intubation, short-term mortality, and changes in PaCO_2_ and pH, whereas composite treatment failure, changes in PaO_2_, and respiratory rate were supported by low-certainty evidence.

Evidence derived exclusively from observational studies was consistently rated as low certainty across all evaluated outcomes. Overall, downgrading was primarily attributable to inconsistency, indirectness in selected outcomes, and imprecision when CI encompassed clinically important differences.

### 3.5 Primary outcome

**FIGURE 2:**
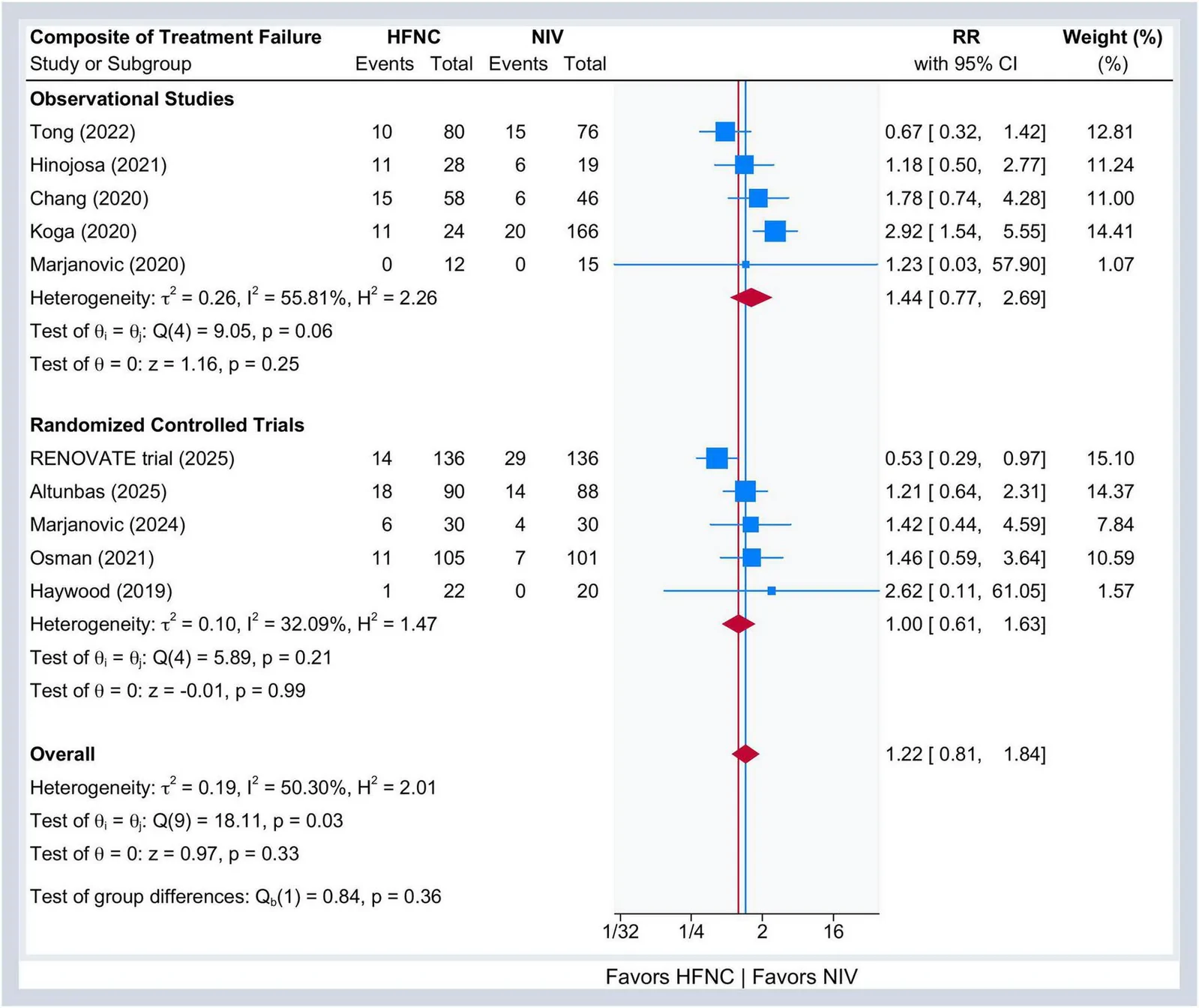
Forest plot of composite outcome of treatment failure stratified by study design. Forest plot comparing the composite outcome of treatment failure between high-flow nasal cannula (HFNC) and noninvasive ventilation (NIV) in adult patients with acute heart failure (AHF)–related acute respiratory failure (ARF), stratified by study design (randomized controlled trials and observational studies). HFNC, high-flow nasal cannula; NIV, noninvasive ventilation; AHF, acute heart failure; ARF, acute respiratory failure; RR, risk ratio; CI, confidence interval.

A total of 10 studies were included in the analysis of the composite outcome of treatment failure comparing HFNC versus NIV. The pooled analysis demonstrated no statistically significant difference between groups (RR = 1.22, 95% CI: 0.81 to 1.84, *p* = 0.33; I^2^ = 50.30%, *p* = 0.03). Subgroup analysis by study design showed consistent non-significant findings across both RCTs (RR = 1.00, 95% CI: 0.61 to 1.63, *p* = 0.99; I^2^ = 32.09%) and observational studies (RR = 1.44, 95% CI: 0.77 to 2.69, *p* = 0.25; I^2^ = 55.81%), with no significant difference between subgroups (*p* = 0.36) (Figure 2).

Leave-one-out sensitivity analysis showed that the pooled estimate remained statistically non-significant after omitting individual studies, except for exclusion of the RENOVATE trial (20), which shifted the estimate toward a statistically significant increase in treatment failure with HFNC (RR = 1.43, 95% CI: 1.02 to 2.00, *p* = 0.036; Supplementary Figure 2).

An additional restricted analysis excluding the post-extubation cohort by Chang et al. (26) and highly unbalanced observational cohort by Koga et al. (23) yielded a similar non-significant result (RR = 0.91, 95% CI: 0.67 to 1.24, *p* = 0.56), with no observed heterogeneity (I^2^ = 0.00%, *p* = 0.44), supporting the overall non-significant interpretation after removal of these clinically distinct or methodologically influential cohorts (Supplementary Figure 3).

Visual inspection of the funnel plot suggested approximate symmetry, with no strong evidence of publication bias (Supplementary Figure 4).

The Galbraith plot identified the RENOVATE trial (20) as a potential outlier, deviating from the regression line, suggesting it may be a contributor to the observed heterogeneity. In contrast, the remaining studies clustered more consistently around the line of no effect (Supplementary Figure 5).

### 3.6 Secondary outcomes

#### 3.6.1 Secondary clinical outcomes

**FIGURE 3:**
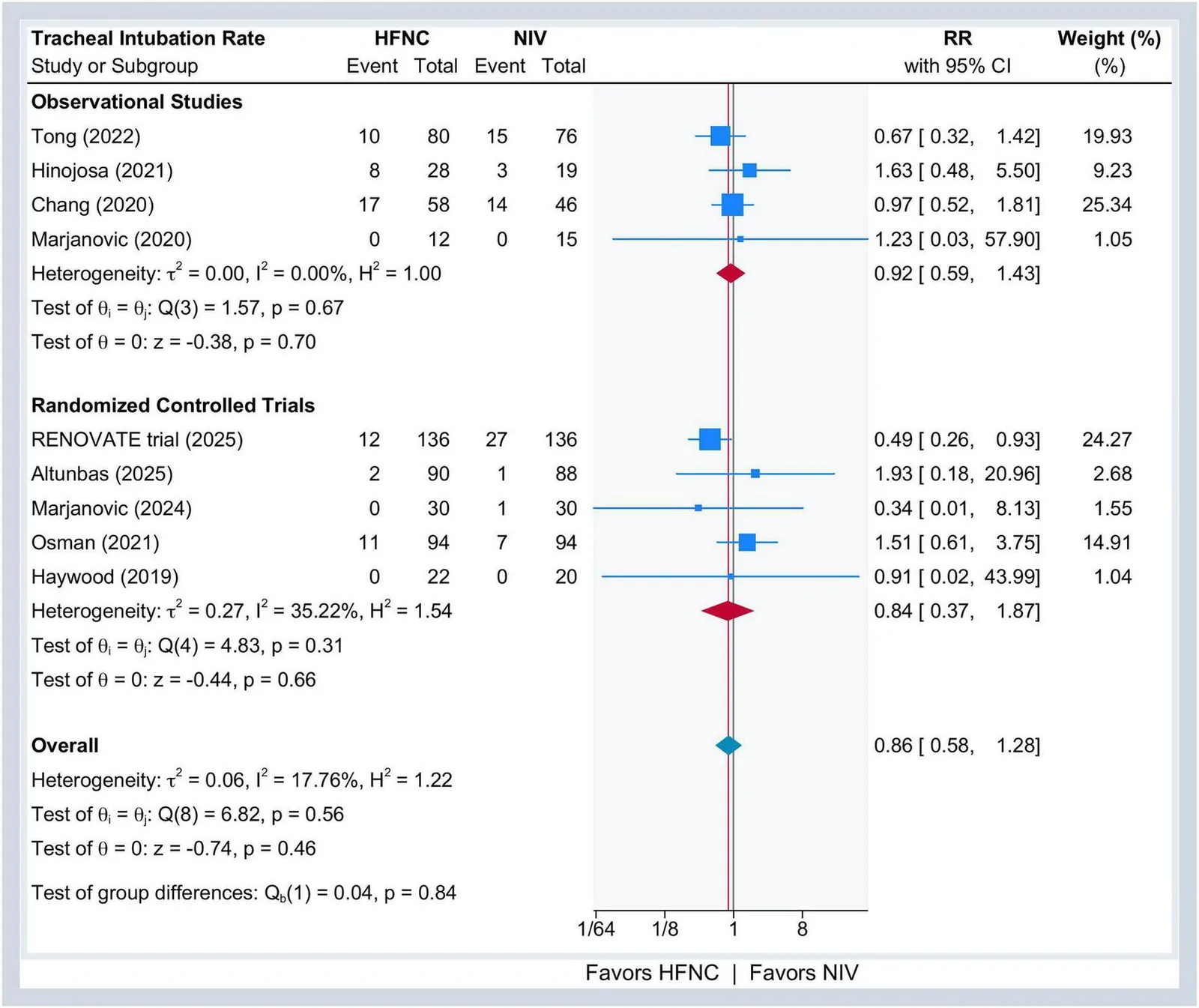
Forest plot of tracheal intubation rate. Forest plot comparing tracheal intubation rate between high-flow nasal cannula (HFNC) and noninvasive ventilation (NIV) in adult patients with acute heart failure (AHF)–related acute respiratory failure (ARF), stratified by study design (randomized controlled trials and observational studies), using a random-effects model. HFNC, high-flow nasal cannula; NIV, noninvasive ventilation; AHF, acute heart failure; ARF, acute respiratory failure; RR, risk ratio; CI, confidence interval.

The pooled analysis of tracheal intubation rate showed no statistically significant difference between HFNC and NIV (RR = 0.86, 95% CI: 0.58 to 1.28, *p* = 0.46), with low heterogeneity (I^2^ = 17.8%). Subgroup analysis demonstrated consistent non-significant findings in observational studies (RR = 0.92, 95% CI: 0.59 to 1.43, *p* = 0.70; I^2^ = 0%) and RCTs (RR = 0.84, 95% CI: 0.37 to 1.87, *p* = 0.66; I^2^ = 35.22%) (Figure 3).

**FIGURE 4:**
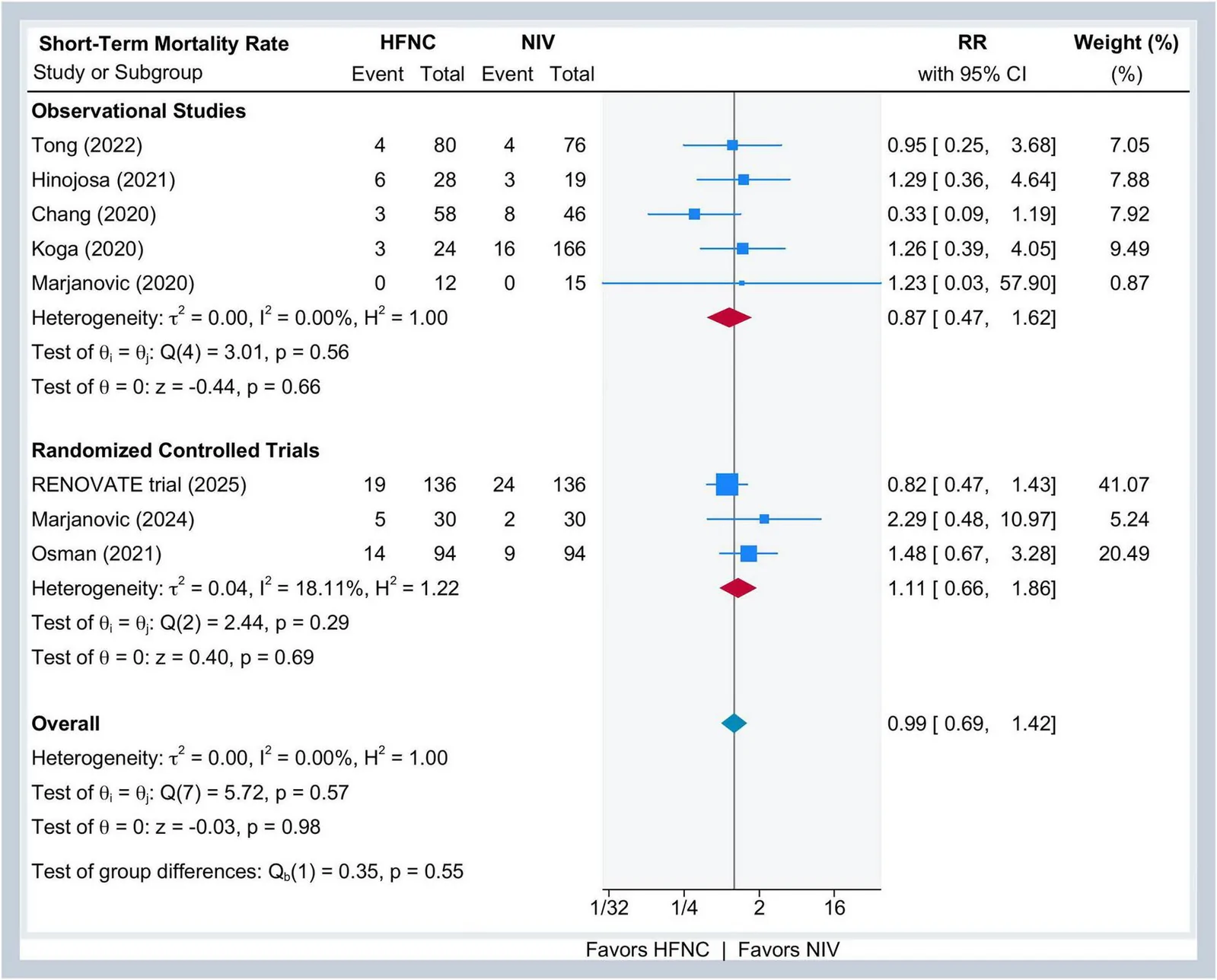
Forest plot of short-term mortality. Forest plot comparing short-term mortality between high-flow nasal cannula (HFNC) and noninvasive ventilation (NIV) in adult patients with acute heart failure (AHF)–related acute respiratory failure (ARF), stratified by study design (randomized controlled trials and observational studies), using a random-effects model. HFNC, high-flow nasal cannula; NIV, noninvasive ventilation; AHF, acute heart failure; ARF, acute respiratory failure; RR, risk ratio; CI, confidence interval.

Similarly, overall short-term mortality demonstrated no statistically significant difference between both groups (RR = 0.99, 95% CI: 0.69 to 1.42, *p* = 0.98), with no heterogeneity (I^2^ = 0%). Subgroup analysis also showed no significant difference in observational studies (RR = 0.87, 95% CI: 0.47 to 1.62, *p* = 0.66; I^2^ = 0%) and RCTs (RR = 1.11, 95% CI: 0.66 to 1.86, *p* = 0.69; I^2^ = 18.1%) (Figure 4).

#### 3.6.2 Arterial blood gas (ABG)

The pooled analysis demonstrated no statistically significant difference in the change of PaO_2_ from baseline between the two strategies (MD = 0.48, 95% CI: −15.57 to 16.53, *p* = 0.95; I^2^ = 79.5%). Subgroup analysis revealed a significant improvement favoring HFNC in observational studies (two studies; MD = 5.82, 95% CI: 2.81 to 8.83, *p* < 0.001; I^2^ = 0%), whereas no statistically significant difference was observed in RCTs (MD = 4.16, 95% CI: −26.83 to 35.16, *p* = 0.79; I^2^ = 80.0%), with no significant subgroup interaction (*p* = 0.92) (Supplementary Figure 6).

Similarly, the change in PaCO_2_ showed no statistically significant difference between both groups (MD = 0.51, 95% CI: −1.67 to 2.70, *p* = 0.65; I^2^ = 36.97%). Subgroup analysis demonstrated consistent non-significant findings across observational studies (MD = −0.62, 95% CI: −8.78 to 7.53, *p* = 0.88; I^2^ = 80.5%) and RCTs (MD = 0.30, 95% CI: −1.27 to 1.86, *p* = 0.71; I^2^ = 0%) (Supplementary Figure 7).

Regarding change in PH from baseline, our pooled analysis demonstrated no statistically significant difference between the two strategies (MD = 0.02, 95% CI: −0.01 to 0.05, *p* = 0.20; I^2^ = 76.9%). Subgroup analysis revealed a significant improvement favoring HFNC in observational studies (two studies; MD = 0.08, 95% CI: 0.02 to 0.14, *p* = 0.01; I^2^ = 43.5%), whereas no statistically significant difference was observed in RCTs (MD = −0.001, 95% CI: −0.01 to 0.01, *p* = 0.85; I^2^ = 0%) (Supplementary Figure 8).

#### 3.6.3 Change from baseline in respiratory rate

Pooled analysis of respiratory rate change from baseline demonstrated no statistically significant difference between HFNC and NIV (MD = −0.08, 95% CI: −2.09 to 1.94, *p* = 0.94; I^2^ = 83.2%). Subgroup analysis showed no significant effect in observational studies (MD = −2.02, 95% CI: −4.23 to 0.18, *p* = 0.07; I^2^ = 8.19%) and RCTs (MD = 0.43, 95% CI: −1.84 to 2.70, *p* = 0.71; I^2^ = 84.8%) (Supplementary Figure 9).

#### 3.6.4 Dyspnea

The pooled analysis of dyspnea scores demonstrated no statistically significant difference between HFNC and NIV (MD = −0.10, 95% CI: −0.55 to 0.35, *p* = 0.66; I^2^ = 0%) (Supplementary Figure 10).

#### 3.6.5 Hospital length of stay

The pooled analysis of hospital length of stay in days demonstrated no statistically significant difference between HFNC and NIV (MD = −1.08 days, 95% CI: −3.13 to 0.96, *p* = 0.30), with no heterogeneity (I^2^ = 0%) (Supplementary Figure 11).

## 4 Discussion

In this meta-analysis, we included 10 studies comprising 1,282 patients comparing HFNC and NIV in patients with AHF-related respiratory failure. Overall, the pooled estimates showed no statistically significant differences between HFNC and NIV in treatment failure, tracheal intubation, or short-term mortality, with consistent findings across the RCT and observational-study subgroups. For ABG parameters, the overall pooled analyses showed no statistically significant differences in changes from baseline in PaO_2_, PaCO_2_, or pH. However, the observational-study subgroup, based on pooled data from only two studies, showed significant improvements in PaO_2_ and pH favoring HFNC, whereas the RCT subgroup showed no statistically significant differences. Similarly, changes from baseline in respiratory rate, along with dyspnea scores and hospital length of stay, also showed no statistically significant differences.

The absence of statistically significant differences between HFNC and NIV in treatment failure, tracheal intubation, and short-term mortality may reflect the ability of both therapies to limit clinical deterioration through distinct physiological pathways (28). NIV delivers constant, high positive end-expiratory pressure (PEEP) and inspiratory pressure support, which directly reduces left ventricular afterload, decreases right ventricular preload, and mechanically unloads the respiratory muscles to improve cardiac output (29, 30). Conversely, while HFNC generates a lower and more variable PEEP (approximately 2–5 cmH_2_O), it compensates by delivering heated, humidified oxygen at high flow rates that precisely match the patient’s elevated inspiratory demand (31–33).

Furthermore, the lack of difference in treatment failure rates may be heavily influenced by patient tolerance (34). NIV frequently induces claustrophobia and patient-ventilator asynchrony, which can trigger a sympathetic surge that exacerbates cardiopulmonary decompensation and leads to therapy failure (35). HFNC utilizes a well-tolerated nasal interface that minimizes this agitation, which may partly offset the greater mechanical support provided by NIV through improved patient tolerance and adherence (36). Although these findings are consistent with the previous meta-analysis by Marjanovic et al. (12), our study extends the evidence base by including the recently published RENOVATE trial (20) and Altunbas et al. (27), increasing the dataset to 10 studies and 1,282 patients, including five RCTs. It also evaluates randomized and observational evidence separately, incorporates a formal GRADE assessment, reports study-specific definitions of treatment failure, and examines the influence of clinically distinct and unbalanced cohorts. With the inclusion of these trials, the RCT estimate for treatment failure moved to the line of no effect (RR = 1.00, 95% CI: 0.61 to 1.63), while the overall primary estimate remained non-significant (RR = 1.22, 95% CI: 0.81 to 1.84). Although heterogeneity in the overall primary analysis was moderate (I^2^ = 50.30%), it disappeared in the restricted analysis after excluding the post-extubation cohort and the highly unbalanced observational cohort (RR = 0.91, 95% CI: 0.67 to 1.24; I^2^ = 0.00%), without changing the statistical significance of the pooled estimate.

When evaluating ABG parameters, our analysis of RCTs demonstrated no significant differences in the changes from baseline for PaO_2_, PaCO_2_, and pH between HFNC and NIV, suggesting that both devices may improve gas exchange through different mechanisms (27). NIV improves gas exchange primarily by reversing alveolar fluid accumulation and recruiting collapsed alveoli through high intrathoracic pressure (37). HFNC, on the other hand, effectively improves PaO_2_ through the reliable delivery of high FiO_2_ combined with mild alveolar recruitment from flow-generated PEEP (31).

Furthermore, the absence of statistically significant differences between the two groups in PaCO_2_ and pH, may be related to the continuous washout of anatomical dead space provided by HFNC, whereas NIV improves ventilation through pressure support (33, 38).

Our ABG findings align with the previous meta-analysis by Marjanovic et al. (12), which similarly reported no statistically significant differences in gas-exchange parameters in the acute setting.

In terms of symptomatic relief, measured by changes in respiratory rate and dyspnea scores, our results showed no significant difference between the two interventions. The reduction in respiratory rate and dyspnea intensity serves as a primary clinical marker for the relief of respiratory distress (39). NIV directly diminishes the work of breathing through inspiratory pressure support, which reduces diaphragmatic effort (40). HFNC achieves a similar reduction in respiratory drive by fulfilling the patient’s high inspiratory flow requirements and reducing the metabolic cost of breathing through the provision of warmed and humidified gas (41). Consequently, both therapies may reduce respiratory distress through distinct physiological mechanisms (27). This lack of statistically significant difference in symptomatic and clinical relief is consistent with the results reported by Marjanovic et al. (12), indicating that neither device showed clear superiority in accelerating clinical recovery from dyspnea.

Finally, our pooled analysis demonstrated no significant difference in the hospital length of stay between patients treated with HFNC and those treated with NIV. This may reflect the lack of detectable between-group differences in intubation, gas exchange, and acute dyspnea. The overall length of stay in AHF patients is more likely dictated by the management of the underlying cardiac pathology, the response to intravenous diuresis protocols, and baseline comorbidities, rather than the initial noninvasive respiratory interface chosen in the emergency department or intensive care unit (42). This finding is consistent with the prior meta-analysis, suggesting that the initial respiratory support modality may not independently determine the overall duration of hospitalization (12).

### 4.1 Strengths and limitations

This meta-analysis has several important strengths. First, we focused primarily on patients with respiratory failure secondary to AHF or ACPE, while also including one predefined post-extubation cohort of patients with established heart failure. By excluding mixed populations without separately extractable heart-failure-specific data, we reduced unrelated clinical heterogeneity and ensured that our findings remained clinically relevant to a cardiogenic population. Second, we included recent RCTs with relatively large sample sizes, which improved the reliability and relevance of our results. Third, we performed subgroup analyses based on study design (RCTs versus observational studies). This approach allowed us to assess the consistency of the findings across different levels of evidence, and the overall results remained similar in both subgroups.

However, several limitations should be considered. Blinding was generally not feasible because of the nature of HFNC and NIV devices (20, 27), which may have influenced decisions regarding treatment escalation, crossover, or intubation (25). Due to inconsistent reporting of subgroup-specific outcomes for hypercapnia status and pulmonary edema severity, as well as variation in NIV mode and interface (CPAP, BiPAP, or helmet CPAP) and HFNC flow and FiO_2_ settings, we were unable to perform subgroup analyses for these factors. This limited our ability to explore clinical heterogeneity and determine whether treatment effects varied across patient and intervention characteristics and may also have affected the pooled estimates. Differences in clinical context, including acute presentations, separately reported subgroups from broader acute respiratory failure cohorts, and a post-extubation heart-failure cohort, may have introduced additional indirectness and clinical heterogeneity. Crossover between treatment arms occurred in several studies and may have reduced the observed differences between the two strategies, particularly in intention-to-treat analyses. Follow-up durations also varied, and short follow-up periods may not have captured delayed deterioration, intubation, or mortality. Finally, some studies relied on an initial clinical diagnosis of AHF, while others excluded patients with marked hypercapnia or hemodynamic instability, limiting the generalizability of the findings to critically ill patients.

### 4.2 Clinical implications and advances in knowledge

Our findings suggest that HFNC may be considered as a tolerance-driven alternative for selected patients with AHF-related respiratory failure who cannot tolerate NIV. Its use should be accompanied by close clinical monitoring and prompt access to NIV escalation if respiratory status fails to improve or deteriorates. This is particularly relevant for patients who cannot tolerate NIV masks due to discomfort, claustrophobia, or poor fit. Nevertheless, NIV should remain readily available as rescue therapy and may be preferred in patients with severe hypercapnia, marked acidosis, or hemodynamic instability. Several studies reported crossover from HFNC to NIV due to lack of efficacy, indicating that a subset of patients still requires the higher and more consistent positive pressure provided by NIV.

Future research should focus on identifying which patients are most likely to benefit from HFNC versus NIV. Studies are needed to evaluate whether baseline factors, such as the severity of hypercapnia, degree of cardiac dysfunction, or early changes in respiratory rate, can predict treatment success or failure. In addition, future trials should improve methodological consistency. Standardizing HFNC settings and NIV modes would reduce variability and allow for more accurate comparisons. Direct comparisons between HFNC and specific NIV modalities, such as CPAP or helmet CPAP, are also needed. In addition, studies should move beyond short-term outcomes. Longer follow-up and more patient-centered outcomes, such as 30-day mortality, hospital readmission, and patient comfort, are essential to better define the clinical role of HFNC in patients with AHF and ACPE.

## 5 Conclusion

In patients with AHF-related respiratory failure, the available evidence showed no statistically significant differences between HFNC and NIV in treatment failure, endotracheal intubation, or short-term mortality. These findings were consistent across RCT and observational-study subgroup analyses. HFNC may be considered as a tolerance-driven alternative in selected patients who cannot tolerate NIV. However, HFNC should be used with close clinical monitoring, and NIV should remain readily available for prompt escalation when clinical improvement is inadequate or deterioration occurs. Further adequately powered multicentered RCTs are needed to better define clinically important differences and identify patients most likely to benefit from each strategy.
