# Supplementary material for: High-flow nasal cannula versus noninvasive ventilation in acute heart failure–related respiratory failure: a systematic review and meta-analysis
Source: Front Med (Lausanne). 2026 Sep 9;13:1941162. doi: 10.3389/fmed.2026.1941162 (PMC13597827; doi:10.3389/fmed.2026.1941162)
Supplement: Supplementary file 1 [file Supplementary_file_1.docx]

**Supplementary Materials**

[Supplementary Figure 1. Risk of bias assessment of randomized controlled trials using ROB-2.	3](#_Toc236980701)

[Supplementary Figure 2. Leave-one-out sensitivity analysis of primary outcome (composite of treatment failure).	4](#_Toc236980702)

[Supplementary Figure 3. Forest plot of composite treatment failure stratified by study design after exclusion of the post-extubation cohort and the highly unbalanced observational cohort.	5](#_Toc236980703)

[Supplementary Figure 4. Funnel plot of composite outcome of treatment failure.	6](#_Toc236980704)

[Supplementary Figure 5. Galbraith plot of composite outcome of treatment failure.	7](#_Toc236980705)

[Supplementary Figure 6. Forest plot of change from baseline in PaO₂.	8](#_Toc236980706)

[Supplementary Figure 7. Forest plot of change from baseline in PaCO₂.	9](#_Toc236980707)

[Supplementary Figure 8. Forest plot of change from baseline in pH.	10](#_Toc236980708)

[Supplementary Figure 9. Forest plot of change from baseline in respiratory rate.	11](#_Toc236980709)

[Supplementary Figure 10. Forest plot of change from baseline in dyspnea score.	12](#_Toc236980710)

[Supplementary Figure 11. Forest plot of change from baseline in hospital length of stay.	13](#_Toc236980711)

[Supplementary Table 1. Detailed search strategy for each database.	14](#_Toc236980712)

[Supplementary Table 2. Definitions of Treatment Failure Across Included Studies.	17](#_Toc236980713)

[Supplementary Table 3. Quality assessment of included observational studies using the Newcastle–Ottawa Scale (NOS).	18](#_Toc236980714)

[Supplementary Table 4. Grading of Recommendations Assessment, Development and Evaluation (GRADE) evidence profile.	19](#_Toc236980715)

# Supplementary Figure 1. Risk of bias assessment of randomized controlled trials using ROB-2.

Risk of bias assessment of included randomized controlled trials using the Cochrane Risk of Bias 2 (RoB-2) tool.

# Supplementary Figure 2. Leave-one-out sensitivity analysis of primary outcome (composite of treatment failure).

Leave-one-out sensitivity analysis assessing the robustness of the pooled estimate for the composite outcome of treatment failure.

# Supplementary Figure 3. Forest plot of composite treatment failure stratified by study design after exclusion of the post-extubation cohort and the highly unbalanced observational cohort.

Forest plot comparing composite treatment failure between high-flow nasal cannula (HFNC) and noninvasive ventilation (NIV) after excluding the post-extubation heart-failure cohort reported by Chang et al. and the markedly unbalanced observational cohort reported by Koga et al. Estimates were stratified by study design and pooled using a random-effects model.

**HFNC =** High-Flow Nasal Cannula**; NIV =** Noninvasive Ventilation**; AHF =** Acute Heart Failure**; ARF =** Acute Respiratory Failure**; RR =** Risk Ratio**; CI =** Confidence Interval

# Supplementary Figure 4. Funnel plot of composite outcome of treatment failure.

Funnel plot assessing potential publication bias for the composite outcome of treatment failure.

# Supplementary Figure 5. Galbraith plot of composite outcome of treatment failure.

Galbraith plot used to explore heterogeneity and identify potential outlier studies contributing to variability in the composite outcome of treatment failure.

# Supplementary Figure 6. Forest plot of change from baseline in PaO₂.

Forest plot comparing change from baseline in arterial partial pressure of oxygen (PaO₂) between high-flow nasal cannula (HFNC) and noninvasive ventilation (NIV) in adult patients with acute heart failure (AHF)–related acute respiratory failure (ARF), stratified by study design (randomized controlled trials and observational studies), using a random-effects model.

**HFNC =** High-Flow Nasal Cannula**; NIV =** Noninvasive Ventilation**; PaO₂ =** Partial Pressure of Arterial Oxygen**; MD =** Mean Difference**; CI =** Confidence Interval

# Supplementary Figure 7. Forest plot of change from baseline in PaCO₂.

Forest plot comparing change in arterial partial pressure of carbon dioxide (PaCO₂) between high-flow nasal cannula (HFNC) and noninvasive ventilation (NIV) in adult patients with acute heart failure (AHF)–related acute respiratory failure (ARF), stratified by study design (randomized controlled trials and observational studies), using a random-effects model.

**HFNC =** High-Flow Nasal Cannula**; NIV =** Noninvasive Ventilation**; PaCO₂ =** Partial Pressure of Arterial Carbon Dioxide**; MD =** Mean Difference**; CI =** Confidence Interval

# Supplementary Figure 8. Forest plot of change from baseline in pH.

Forest plot comparing change in arterial pH between high-flow nasal cannula (HFNC) and noninvasive ventilation (NIV) in adult patients with acute heart failure (AHF)–related acute respiratory failure (ARF), stratified by study design (randomized controlled trials and observational studies), using a random-effects model.

**HFNC =** High-Flow Nasal Cannula**; NIV =** Noninvasive Ventilation**; MD =** Mean Difference**; CI =** Confidence Interval

# Supplementary Figure 9. Forest plot of change from baseline in respiratory rate.

Forest plot comparing change in respiratory rate (RR) between high-flow nasal cannula (HFNC) and noninvasive ventilation (NIV) in adult patients with acute heart failure (AHF)–related acute respiratory failure (ARF), stratified by study design (randomized controlled trials and observational studies), using a random-effects model.

**HFNC =** High-Flow Nasal Cannula**; NIV =** Noninvasive Ventilation**; PaCO₂ =** Partial Pressure of Arterial Carbon Dioxide**; MD =** Mean Difference**; CI =** Confidence Interval

# Supplementary Figure 10. Forest plot of change from baseline in dyspnea score.

Forest plot comparing dyspnea score between high-flow nasal cannula (HFNC) and noninvasive ventilation (NIV) in adult patients with acute heart failure (AHF)–related acute respiratory failure (ARF) using a random-effects model.

**HFNC =** High-Flow Nasal Cannula**; NIV =** Noninvasive Ventilation**; PaCO₂ =** Partial Pressure of Arterial Carbon Dioxide**; MD =** Mean Difference**; CI =** Confidence Interval

# Supplementary Figure 11. Forest plot of change from baseline in hospital length of stay.

Forest plot comparing hospital length of stay between high-flow nasal cannula (HFNC) and noninvasive ventilation (NIV) in adult patients with acute heart failure (AHF)–related acute respiratory failure (ARF) using a random-effects model.

**HFNC =** High-Flow Nasal Cannula**; NIV =** Noninvasive Ventilation**; PaCO₂ =** Partial Pressure of Arterial Carbon Dioxide**; MD =** Mean Difference**; CI =** Confidence Interval.

# Supplementary Table 1. Detailed search strategy for each database.

| Database | Search term | Filter | Results |
| --- | --- | --- | --- |
| PubMed | ("Heart Failure"[Mesh] OR "Pulmonary Edema"[Mesh] OR "Acute Heart Failure" OR "Heart Decompensation" OR "Cardiac Failure" OR "Congestive Heart Failure" OR "Decompensated Heart Failure" OR "Acute Decompensated Heart Failure" OR ADHF OR AHF OR "Pulmonary Edema"[Mesh] OR "Cardiogenic Pulmonary Edema" OR "Acute Pulmonary Edema")  **AND**  ("High-Flow Nasal Cannula"[Mesh] OR "High Flow Nasal Cannula" OR HFNC OR "High-Flow Oxygen" OR "High Flow Oxygen Therapy" OR "Nasal High Flow" OR NHF OR "Heated Humidified High Flow" OR "Heated Humidified Oxygen" OR "High Flow Therapy")  **AND**  ("Noninvasive Ventilation"[Mesh] OR "Noninvasive Positive Pressure Ventilation" OR "Non-invasive Ventilation" OR NIV OR NIPPV OR "Positive Pressure Ventilation" OR "Continuous Positive Airway Pressure"[Mesh]OR "Continuous Positive Airway Pressure" OR CPAP OR "Bilevel Positive Airway Pressure" OR BiPAP OR "Noninvasive Respiratory Support") | All Filed | **61** |
| Scopus | ("Heart Failure" OR "Pulmonary Edema" OR "Acute Heart Failure" OR "Heart Decompensation" OR "Cardiac Failure" OR "Congestive Heart Failure" OR "Decompensated Heart Failure" OR "Acute Decompensated Heart Failure" OR ADHF OR AHF OR "Pulmonary Edema" OR "Cardiogenic Pulmonary Edema" OR "Acute Pulmonary Edema")  **AND**  ("High-Flow Nasal Cannula" OR "High Flow Nasal Cannula" OR HFNC OR "High-Flow Oxygen" OR "High Flow Oxygen Therapy" OR "Nasal High Flow" OR NHF OR "Heated Humidified High Flow" OR "Heated Humidified Oxygen" OR "High Flow Therapy")  **AND**  ("Noninvasive Ventilation" OR "Noninvasive Positive Pressure Ventilation" OR "Non-invasive Ventilation" OR NIV OR NIPPV OR "Positive Pressure Ventilation" OR "Continuous Positive Airway Pressure" OR CPAP OR "Bilevel Positive Airway Pressure" OR BiPAP OR "Noninvasive Respiratory Support") | Article title, abstract, keywords | **363** |
| Web of Science | ("Heart Failure" OR "Pulmonary Edema" OR "Acute Heart Failure" OR "Heart Decompensation" OR "Cardiac Failure" OR "Congestive Heart Failure" OR "Decompensated Heart Failure" OR "Acute Decompensated Heart Failure" OR ADHF OR AHF OR "Pulmonary Edema" OR "Cardiogenic Pulmonary Edema" OR "Acute Pulmonary Edema")  **AND**  ("High-Flow Nasal Cannula" OR "High Flow Nasal Cannula" OR HFNC OR "High-Flow Oxygen" OR "High Flow Oxygen Therapy" OR "Nasal High Flow" OR NHF OR "Heated Humidified High Flow" OR "Heated Humidified Oxygen" OR "High Flow Therapy")  **AND**  ("Noninvasive Ventilation" OR "Noninvasive Positive Pressure Ventilation" OR "Non-invasive Ventilation" OR NIV OR NIPPV OR "Positive Pressure Ventilation" OR "Continuous Positive Airway Pressure" OR CPAP OR "Bilevel Positive Airway Pressure" OR BiPAP OR "Noninvasive Respiratory Support") | All Filed | **118** |
| Cochrane library | ("Heart Failure" OR "Pulmonary Edema" OR "Acute Heart Failure" OR "Heart Decompensation" OR "Cardiac Failure" OR "Congestive Heart Failure" OR "Decompensated Heart Failure" OR "Acute Decompensated Heart Failure" OR ADHF OR AHF OR "Pulmonary Edema" OR "Cardiogenic Pulmonary Edema" OR "Acute Pulmonary Edema")  **AND**  ("High-Flow Nasal Cannula" OR "High Flow Nasal Cannula" OR HFNC OR "High-Flow Oxygen" OR "High Flow Oxygen Therapy" OR "Nasal High Flow" OR NHF OR "Heated Humidified High Flow" OR "Heated Humidified Oxygen" OR "High Flow Therapy")  **AND**  ("Noninvasive Ventilation" OR "Noninvasive Positive Pressure Ventilation" OR "Non-invasive Ventilation" OR NIV OR NIPPV OR "Positive Pressure Ventilation" OR "Continuous Positive Airway Pressure" OR CPAP OR "Bilevel Positive Airway Pressure" OR BiPAP OR "Noninvasive Respiratory Support") | All Filed | **60** |

# Supplementary Table 2. Definitions of Treatment Failure Across Included Studies.

| Study (First Author, Year) | Definition of "Treatment Failure" utilized in the primary pooled analysis |
| --- | --- |
| Altunbas (2025) | Composite outcome occurring within 72 hours, including the need for intubation or treatment switch due to hypoxemia, hypercapnia, excessive breathing effort, cardiac arrest, or loss of airway protection. |
| Maia (2024) [RENOVATE] | Composite outcome defined strictly as the need for endotracheal intubation or death from any cause within 7 days of randomization. |
| Marjanovic (2024) | Composite criterion including premature termination of treatment before 1 hour, and/or death within 8 days, and/or endotracheal intubation within 8 days. |
| Osman (2021) | Escalation to endotracheal intubation based on predefined clinical criteria (intolerance to therapy, worsening respiratory failure, hemodynamic instability, or deterioration of consciousness). |
| Haywood (2019) | Requirement for endotracheal intubation or subjective crossover to the alternative therapy occurring within the first 72 hours of enrolment. |
| Tong (2022) | The requirement for endotracheal intubation during the hospital stay. |
| Hinojosa (2021) | Combined objective endpoint of the need for orotracheal intubation or change of therapy due to respiratory worsening. |
| Chang (2020) | Occurring within 72 hours. Defined in the HFNC group as escalation to NIV or invasive mechanical ventilation (IMV); defined in the NIV group as the need for IMV. |
| Koga (2020) | Composite outcome including: (i) endotracheal intubation, (ii) switching to another treatment without improvement, or (iii) death during HFNC or NIV. |
| Marjanovic (2020) | Need for endotracheal intubation or urgent escalation to invasive mechanical ventilation. |

# Supplementary Table 3. Quality assessment of included observational studies using the Newcastle–Ottawa Scale (NOS).

| **study ID** | **Selection** | | | | **Comparability** | **Outcome** | | |  |
| --- | --- | --- | --- | --- | --- | --- | --- | --- | --- |
|  | Representativeness of the exposed cohort | Selection of the non-exposed cohort | Ascertainment of exposure | Outcome of interest was not present at start of study | Comparability of Cohorts | Assessment of outcome | Long Enough Follow-up | Adequacy of follow up of cohorts | Overall |
| **Chang 2020** | ★ | ★ | ★ | ★ | ★★ | ★ | ★ | ★ | **Good** |
| **Hinojosa 2021** | ★ | ★ | ★ | ★ | ★ | ★ | ★ | ★ | **Good** |
| **Koga 2020** | ★ | ★ | ★ | ★ | ★★ | ★ | ★ | ★ | **Good** |
| **Marjanovic 2020** | ★ | ★ | ★ | ★ |  | ★ | ★ | ★ | **Poor** |
| **Tong 2022** | ★ | ★ | ★ | ★ | ★ | ★ | ★ | ★ | **Good** |

# Supplementary Table 4. Grading of Recommendations Assessment, Development and Evaluation (GRADE) evidence profile.

| **Certainty assessment** | | | | | | | **No. of patients** | | **Effect** | | **Certainty** | **Importance** |
| --- | --- | --- | --- | --- | --- | --- | --- | --- | --- | --- | --- | --- |
| **No. of studies** | **Study design** | **Risk of bias** | **Inconsistency** | **Indirectness** | **Imprecision** | **Other considerations** | **HFNC** | **NIV** | **Relative (95% CI)** | **Absolute (95% CI)** |  |  |
| **Composite treatment failure** | | | | | | | | | | | | |
| 10 | RCTs + observational | Seriousᵃ | Not seriousᵇ | Seriousᶜ | Not seriousᵈ | No other concerns identifiedᵉ | 585 | 697 | RR 1.22  (0.81 to 1.84) | _ | ⊕⊕◯◯ Low | Critical |
| 5 | RCTs | Not seriousᶠ | Not seriousᵇ | Not seriousᶜ | Seriousᵈ | No other concerns identifiedᵉ | 383 | 375 | RR 1.00  (0.61 to 1.63) | _ | ⊕⊕⊕◯ Moderate | Critical |
| 5 | Observational studies | Not seriousᵍ | Not seriousᵇ | Not seriousᶜ | Not seriousᵈ | No upgrading factors identified | 202 | 322 | RR 1.44  (0.77 to 2.69) | _ | ⊕⊕◯◯ Low | Critical |
| **Tracheal intubation** | | | | | | | | | | | | |
| 9 | RCTs + observational | Not seriousᵃ | Not seriousᵇ | Not serious | Seriousᵈ | Publication bias not assessableᵉ | 550 | 524 | RR 0.86 (0.58 to 1.28) | _ | ⊕⊕⊕◯ Moderate | Critical |
| 5 | RCTs | Not seriousᶠ | Not seriousᵇ | Not serious | Seriousᵈ | Publication bias not assessableᵉ | 372 | 368 | RR 0.84 (0.37 to 1.87) | _ | ⊕⊕⊕◯ Moderate | Critical |
| 4 | Observational studies | Not seriousᵍ | Not seriousᵇ | Not serious | Not seriousᵈ | No upgrading factors identified | 178 | 156 | RR 0.92 (0.59 to 1.43) | _ | ⊕⊕◯◯ Low | Critical |
| **Short-term mortality** | | | | | | | | | | | | |
| 8 | RCTs + observational | Not seriousᵃ | Not seriousᵇ | Not seriousʰ | Seriousᵈ | Publication bias not assessableᵉ | 462 | 582 | RR 0.99 (0.69 to 1.42) | _ | ⊕⊕⊕◯ Moderate | Critical |
| 3 | RCTs | Not seriousᶠ | Not seriousᵇ | Not seriousʰ | Seriousᵈ | Publication bias not assessableᵉ | 260 | 260 | RR 1.11 (0.66 to 1.86) | _ | ⊕⊕⊕◯ Moderate | Critical |
| 5 | Observational studies | Not seriousᵍ | Not seriousᵇ | Not seriousʰ | Not seriousᵈ | No upgrading factors identified | 202 | 322 | RR 0.87 (0.47 to 1.62) | _ | ⊕⊕◯◯ Low | Critical |
| **Change from baseline in PaO₂ (mmHg)** | | | | | | | | | | | | |
| 5 | RCTs + observational | Not seriousᵃ | Seriousᵇ | Not serious | Not seriousᵈ | Publication bias not assessableᵉ | 231 | 231 | _ | MD 0.48 mmHg higher (15.57 lower to 16.53 higher) | ⊕⊕◯◯ Low | Important |
| 3 | RCTs | Not seriousᶠ | Seriousᵇ | Not serious | Not seriousᵈ | Publication bias not assessableᵉ | 139 | 140 | _ | MD 4.16 mmHg higher (26.83 lower to 35.16 higher) | ⊕⊕⊕◯ Moderate | Important |
| 2 | Observational studies | Not seriousᵍ | Not seriousᵇ | Not serious | Not seriousᵈ | No upgrading factors identified | 92 | 91 | _ | MD 5.82 mmHg higher (2.81 to 8.83 higher) | ⊕⊕◯◯ Low | Important |
| **Change from baseline in PaCO₂ (mmHg)** | | | | | | | | | | | | |
| 6 | RCTs + observational | Not seriousᵃ | Not seriousᵇ | Not serious | Seriousᵈ | Publication bias not assessableᵉ | 312 | 310 | _ | MD 0.51 mmHg higher (1.67 lower to 2.70 higher) | ⊕⊕⊕◯ Moderate | Important |
| 4 | RCTs | Not seriousᶠ | Not seriousᵇ | Not serious | Not seriousᵈ | Publication bias not assessableᵉ | 220 | 219 | _ | MD 0.30 mmHg higher (1.27 lower to 1.86 higher) | ⊕⊕⊕⊕ High | Important |
| 2 | Observational studies | Not seriousᵍ | Not seriousᵇ | Not serious | Not seriousᵈ | No upgrading factors identified | 92 | 91 | _ | MD 0.62 mmHg lower (8.78 lower to 7.53 higher) | ⊕⊕◯◯ Low | Important |
| **Change from baseline in pH** | | | | | | | | | | | | |
| 6 | RCTs + observational | Not seriousᵃ | Seriousᵇ | Not serious | Not seriousᵈ | Significant subgroup interaction; interpreted by design subgroup | 328 | 322 | _ | MD 0.02 higher (0.01 lower to 0.05 higher) | ⊕⊕⊕◯ Moderate | Important |
| 4 | RCTs | Not seriousᶠ | Not seriousᵇ | Not serious | Not seriousᵈ | Publication bias not assessableᵉ | 236 | 231 | _ | MD 0.001 lower (0.01 lower to 0.01 higher) | ⊕⊕⊕⊕ High | Important |
| 2 | Observational studies | Not seriousᵍ | Not seriousᵇ | Not serious | Not seriousᵈ | No upgrading factors identified | 92 | 91 | _ | MD 0.08 higher (0.02 to 0.14 higher) | ⊕⊕◯◯ Low | Important |
| **Change from baseline in respiratory rate (breaths/min)** | | | | | | | | | | | | |
| 6 | RCTs + observational | Not seriousᵃ | Seriousᵇ | Not serious | Not seriousᵈ | Publication bias not assessableᵉ | 327 | 323 | _ | MD 0.08 breaths/min lower (2.09 lower to 1.94 higher) | ⊕⊕◯◯ Low | Important |
| 4 | RCTs | Not seriousᶠ | Seriousᵇ | Not serious | Not seriousᵈ | Publication bias not assessableᵉ | 235 | 232 | _ | MD 0.43 breaths/min higher (1.84 lower to 2.70 higher) | ⊕⊕⊕◯ Moderate | Important |
| 2 | Observational studies | Not seriousᵍ | Not seriousᵇ | Not serious | Not seriousᵈ | No upgrading factors identified | 92 | 91 | _ | MD 2.02 breaths/min lower (4.23 lower to 0.18 higher) | ⊕⊕◯◯ Low | Important |
| **Dyspnea score** | | | | | | | | | | | | |
| 3 | RCTs | Not seriousᶠ | Not seriousᵇ | Seriousⁱ | Not seriousᵈ | Publication bias not assessableᵉ | 191 | 189 | _ | MD 0.10 points lower (0.55 lower to 0.35 higher) | ⊕⊕⊕◯ Moderate | Important |
| **Hospital length of stay (days)** | | | | | | | | | | | | |
| 3 | Observational studies | Not seriousᵍ | Not seriousᵇ | Not serious | Not seriousᵈ | No upgrading factors identified | 166 | 141 | _ | MD 1.08 days lower (3.13 lower to 0.96 higher) | ⊕⊕◯◯ Low | Important |

GRADE evidence profile was prepared according to the GRADE Handbook and GRADEpro recommendations. ᵃ Mixed-design estimates are presented because they were prespecified and reported in the manuscript. They were interpreted as supplementary evidence, with judgments driven by the contribution and limitations of both randomized and observational studies. ᵇ Inconsistency was not rated down solely because of I². Downgrading was applied only when differences in magnitude or direction were considered important and remained unexplained; potential double-counting with imprecision was avoided. ᶜ For composite treatment failure, variation in outcome definitions was considered important in the mixed-design analysis. Within the RCT-only analysis, this variation was not judged sufficient by itself to reduce certainty. ᵈ Imprecision was judged according to whether the confidence interval included meaningfully different clinical conclusions. A single-level downgrade was used when important benefit and harm could not be excluded; wide intervals were not automatically penalized when the clinical interpretation remained unchanged. ᵉ Publication bias could not be reliably assessed for outcomes with fewer than 10 studies. This limitation was recorded but was not used as an automatic reason for downgrading. ᶠ The randomized evidence was not downgraded automatically for isolated study-level concerns. Objective outcomes were considered less vulnerable to lack of blinding, and the body of evidence was downgraded only when limitations were likely to materially affect the pooled estimate. ᵍ Observational evidence started at low certainty. No additional downgrade was applied when the available cohorts were broadly consistent and no major outcome-specific limitation was judged sufficient to reduce confidence further; no upgrading criterion was identified. ʰ Differences among in-hospital, ICU, 28-day, and 30-day mortality were considered compatible with a clinically coherent short-term mortality construct and were therefore not judged seriously indirect. ⁱ Dyspnea evidence was downgraded once for indirectness because instruments and/or assessment timepoints were not sufficiently uniform to ensure a fully common construct.

**Abbreviations: CI**, confidence interval; **HFNC**, high-flow nasal cannula; **MD**, mean difference; **NIV**, noninvasive ventilation; **PaCO**₂, arterial partial pressure of carbon dioxide; **PaO**₂, arterial partial pressure of oxygen; **RCT**, randomized controlled trial; **RR**, risk ratio.
